# The effect of search mode on dimension weighting

**DOI:** 10.3389/fpsyg.2014.01054

**Published:** 2014-10-06

**Authors:** Takatsune Kumada

**Affiliations:** Graduate School of Informatics, Kyoto UniversityKyoto, Japan

**Keywords:** attention, visual search, search mode, frature-based control, response congruency

## Abstract

In a visual feature search task, reaction times to a singleton target are known to be shorter when participants have advance knowledge of the defining-features of targets. The present study examined whether the prior-knowledge effect is influenced by search modes (feature vs. singleton). In addition, using a variant of the flanker task, the present study assessed whether prior-knowledge affected efficiency of attentional focusing to a target. When participants performed a target discrimination task (i.e., compound search task), using a singleton detection mode, no prior-knowledge effect was found (Experiments 1 and 3). However, when the same task was performed using a feature search mode, prior-knowledge facilitated performance (Experiment 2). This suggests that the dimension weighting of a target-defining feature is modulated by the search mode. Also flanker response congruency was affected by prior-knowledge suggesting that the dimension weighting correlated with attentional focusing to targets. On the other hand, inter-trial dimensional priming was not affected by the search mode. Implications for mechanisms of feature-based top-down control of attention in visual feature search are discussed.

## Introduction

Previous studies have reported that prior knowledge of the target-defining feature dimension facilitate detection of a target in visual search tasks (Treisman, 1988; Müller et al., 1995; Found and Müller, 1996). The first demonstration of the prior knowledge effect in visual feature search was provided by Treisman (1988). In one experiment of her study, targets were defined by dimensions of color, orientation, or size in a feature search task. Three prior knowledge conditions were employed. One condition entailed instructing participants that to-be-detected targets involved features that were fixed for a given dimension. For example, a target feature value of blue was fixed for the color dimension within a block of trials (e.g., a blue bar among green bars). In the second prior knowledge condition, targets were defined by varying feature values within a single dimension, i.e., the within-dimension condition. For example, within the color dimension a target feature might assume different values within a trial block (e.g., either blue, red, or white bar among green bars). The third prior knowledge condition, the cross-dimension condition, defined targets as any one of three feature dimensions (color, orientation, or size). For example, a target was either blue vertical bar, green tilted bar, or a green vertical bar which was larger than distractors. It turns out that reaction times (RTs) to targets in the within-dimension condition were the same as those in the fixed-target condition and both were significantly faster than RTs to targets in the cross-dimension condition. The difference between RTs in the cross-dimension condition and those of within-dimension condition was referred to as cross-dimensional cost (CDC) in Müller and Krummenacher (2006). An interpretation of the CDC holds that prior knowledge (i.e., regarding the dimension of change) is responsible for facilitating target detection even when targets were feature singletons in a search display (Treisman, 1988; Müller et al., 1995). That is, because defining dimensions of targets were unspecified in the cross-dimension condition but not in the within-dimension condition, the latter allows for advantageous use of dimensional knowledge in target detection.

A number of previous studies have shown that prior knowledge about the relevant dimension facilitates performance in simple search tasks where participants must detect the mere presence or absence of a target in a search display (e.g., Müller et al., 1995). However, other studies have reported little to no effect of prior dimensional knowledge (Kumada, 2001; Krummenacher et al., 2002); in these cases, the task involved is typically a *compound* search task, namely a task that requires participants to not only identify the presence of single-feature targets but also to assess (and report on) an attribute associated with the target item. For example, Kumada (2001) used a compound search task to examine CDC in within and cross-dimension conditions. In the within-dimension condition, a single feature defined the target such that the orientation (left, right, or horizontal) of a target gray bar distinguished the target relative to surrounding distractors that were vertical gray bars. In the cross-dimension condition, using the same distractors, a target could be any of the following: a gray left-tilted bar, a green vertical bar, or a larger gray vertical bar (relative to distractor sizes). In both conditions, a compound search required that participants not only identify a target, but they also had to report on a small L-shape, oriented to the left or right, that was embedded in the target (as well as in each distractor). In other words, the compound search combined a singleton detection task with an independent response selection task such that participants had to respond to the orientation of L-shape following detection of a singleton target (based on the orientation). In the study, Kumada found no CDC in the compound search task: RTs to targets in the within-dimension condition were equivalent to those in the cross-dimension condition.

The dissociation of results in a simple search task from those in a compound search task was also found in another RT measure. Especially, inter-trial target consistency which is pertinent to the cross-dimension condition. Inter-trial consistency refers to conditional repetition of target-defining features from trial *n* − 1 to trial *n* in the cross-dimension condition. In feature search tasks, the consistency of a given feature from one trial to the next, even if target-defining dimensions are unknown in advance (as in cross-dimension conditions) may offer sufficient prior information to affect RTs (see Kristjánsson and Campana, 2010; Lamy and Kristjánsson, 2013, for reviews). If so, then RTs to targets should be faster when a defining-feature of the current target, i.e., on trial *n*, is the same as that presented on an immediately preceding trial than when the current target feature differs from that on trial *n* − 1; this is referred to as Inter-Trial-Facilitation, ITF (Found and Müller, 1996; Kumada, 2001). So far, it has been reported that the ITF, which is observed in cross-dimension conditions with a feature search, is largely reduced or even eliminated when a compound search is required (Kumada, 2001; Krummenacher et al., 2002; Müller and Krummenacher, 2006; Theeuwes et al., 2006).

The results in the compound search task are counter-intuitive, in light of a two-stage model of visual attention (Treisman and Gelade, 1980; Wolfe, 1994; Itti and Koch, 2001), and it's extension with the dimensional weighting account (Müller et al., 1995). That is, such models assume a final response selection stage is preceded by an initial parallel (spatial) processing stage involving feature selection which is followed by serial search stage which entails focal attending to an attribute extracted from this target. The dimensional weighting account assumed that prior-knowledge of a target's dimension facilitates (or weights) target selection in the first stage. In this case, it seems reasonable that the head start provided to within-dimension conditions in the parallel processing stage should carry-over to the second stage, leading to faster attentional focusing to targets. This advantage of dimensional weighting is observed as CDC. In addition, the dimensional weighting in a current trial is considered to carry-over to the next trial, yielding the ITF (Found and Müller, 1996). Therefore, if both CDC and ITF depend upon a dimensional weighting mechanism in the first parallel feature processing stage, which is activated by prior-knowledge, then it should emerge even in a compound search task. This is because in this task the first stage is the same as the first stage in feature search tasks in that both involve compound search.

In order to account for these counter-intuitive results, it has been proposed that they reflect processing in post-selective stages (i.e., involving target analysis and response selection) (e.g., Cohen and Magen, 1999; Feintuch and Cohen, 2002; Mortier et al., 2005; Theeuwes et al., 2006). Although the particular explanation of results without the dimension weighting account differs according to researcher, the explanation of Theeuwes et al. is particularly relevant here. They proposed that the first stage, involving parallel visual processing, is impenetrable from top-down control (Theeuwes, 1992). This implies that the first stage is irrelevant for the CDC and the ITF. In order to test this, Mortier et al. (2005) used a non-search task in which only one target was present in a display with no accompanying distractors. Despite the fact that no parallel processing for target selection was required in this task, these researchers found both CDC and ITF. They concluded that results, which have been typically attributed to early top-down modulation of dimensional signals in the first pre-attentive stage, may be attributable to later post-attentive processes.

Recently, Rangelov et al. (2012) proposed a new framework which assumes that multiple independent mechanisms are responsible for the inter-trial effect in visual search and related tasks, including non-search tasks. According to this view of multiple-weighting-systems (MWS), they hypothesize that inter-trial effects involve MWS related factors at three levels of processing: (1) stimulus selection, (2) perceptual or semantic analysis of selected stimuli, and (3) response selection. Furthermore, this framework theorized these three systems work independently. Subsequently a series of studies revealed that this framework is able to account for a wide range of results related to the ITF in visual search (Rangelov et al., 2011a,b, 2012).

Compared to the mechanism of ITF in visual search, less is known about the mechanism of CDC is in this process. In this study, the CDC mechanism is further investigated by specifically focusing on conditions in which CDC emerges in a compound search task. Accordingly, I manipulated visual search strategy in a compound search task. Recent studies have documented that search strategy can alter the mode of attentional control, leading performance changes in visual search (Bacon and Egeth, 1994; Leber and Egeth, 2006; Inukai et al., 2010; see also Theeuwes, 2004). Seminal work by Bacon and Egeth (1994) proposed two search modes: the singleton detection mode and the feature search mode. They examined effects of these search modes using a visual feature search task with singleton distractors. In this task, it is well known that visual search is disrupted by presentation of a single visually salient distractor, i.e., a singleton distractor (Theeuwes, 1991, 1992). Bacon and Egeth examined whether the disruption effect depended upon search mode. They hypothesized that disruption was specific to the singleton detection mode in which participants searched for any singletons in a search display in spite of the fact that participants knew the target features in advance. To test this hypothesis, these investigators included multiple targets into displays when participants had to engage in a feature search. Thus, targets were no longer singletons in these search displays. This manipulation succeeded eliminating the disruptive effect of singleton distractors, presumably by encouraging a feature search mode. Moreover, it demonstrated that search mode (feature search vs. singleton detection) affected performance in visual search tasks, via modifying the contribution of top-down control to the first parallel visual processing stage.

The effect of the search strategy on dimensional weighting has been partly investigated in previous studies using a different research context (Lamy et al., 2008; see also Lamy et al., 2006). For instance, in Experiment 1 of Lamy et al. (2008), the ITF was examined using displays with multiple targets (1, 3, and 5) using a compound search task; they assessed RTs as a function of retained number of targets over consecutive trials (the number of consecutive trials with unchanged target numbers varied between zero to four). Results indicated that a sizable ITF occurred only in repeated singleton-target trials. They referred to this phenomenon as “within-dimension singleton priming,” thus implying that the ITF was found when participants could not adopt a singleton detection mode. Although Lamy et al. (2008) reported that one effect related to dimensional weighting, ITF, was modulated by participants' search mode, other measures of dimensional weighting effect, such as CDC, were not examined. Furthermore, this study did not directly manipulate search mode, i.e., singleton detection vs. feature search.

The present study had a primary goal of investigating whether or not the search mode adopted by participants affects their performance in a compound search tasks as it is related to dimensional weighting (i.e., CDC and ITF). Specifically, the hypothesis tested in the present study is that the failure of prior dimension knowledge to facilitate performance in a compound search task arises from the fact that participants adopt the singleton detection mode. That is, if the absence of a dimensional-weighting effect in a compound search task is due to a participant's search mode, then dimensional prior-knowledge of targets will be employed in visual feature search when participants *do not use* the singleton detection mode. Based on this hypothesis, I predicted that the CDC and ITF should emerge if participants are *forced not to adopt* the singleton detection mode. To test this prediction, I employed a display manipulation with multiple targets similar to that of Bacon and Egeth (1994) in a compound search task with multi-dimensional singleton targets.

**Figure 1:**
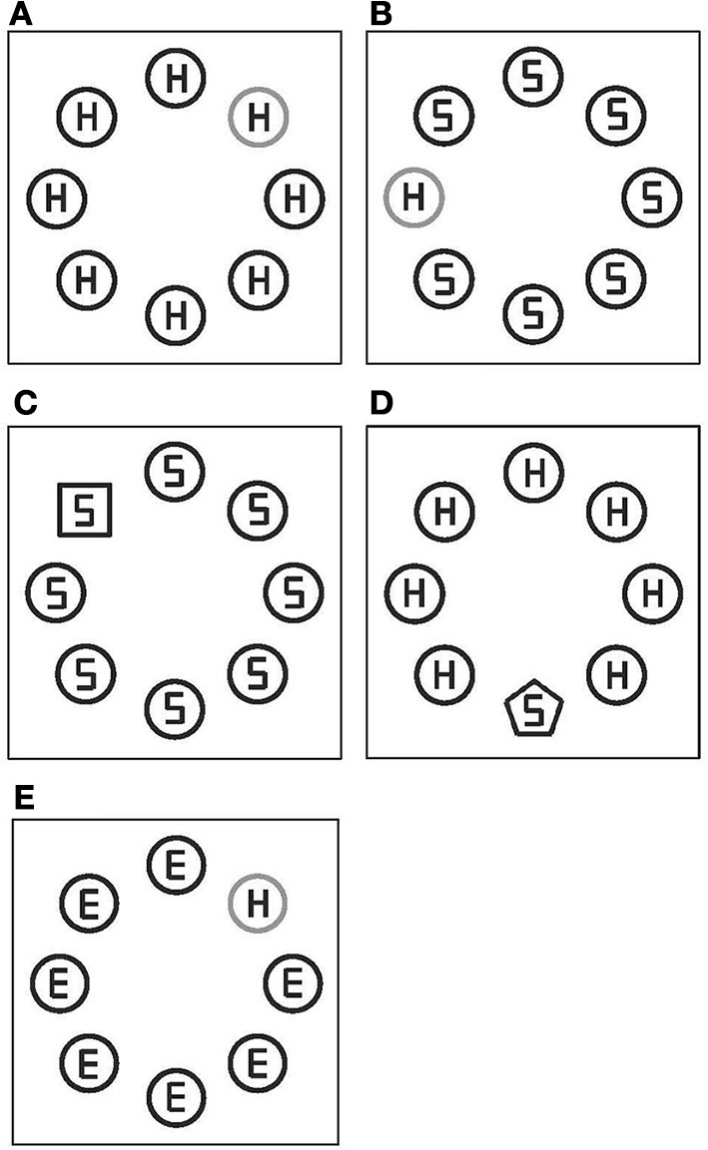
**Examples of stimulus displays in Experiment 1. (A)** Yellowish-red target, **(B)** yellowish-green target, **(C)** square target, **(D)** pentagon target, and **(E)** yellowish-red target, for response congruent trials **(A,C)**, for response incongruent trials **(B,D)**, and for neutral trials **(E)**. All the stimuli were yellow on black background, except for the yellowish-red and yellowish-green target (depicted in gray).

A second goal of the present study involved investigating the effect of dimension weighting on focusing of spatial attention. For this reason, I examined a response congruency measure, which is based on RTs to a target's reported attribute when this attribute was identical (congruent) with distractor attributes vs. when it was not. Specifically, each target contained one of two reported attributes (H or S); in addition, all distractors also contained one of these attributes (see exemplar stimuli in Figure 1). RTs to a reported-attribute in a target were analyzed with respect to congruency of this attribute with attributes present in distractors. This measure permits an examination of the locus in which dimensional knowledge processing occurs. Because the response congruency effect is assumed to reflect the degree of attentional focusing on targets (Eriksen and St. James, 1986; Theeuwes and Van der Burg, 2011), it is possible to assess whether top-down dimensional knowledge affects guidance of spatial attention.

The third goal of this study was to investigate whether CDD is caused by inflated RTs to targets in the cross-dimension block resulting from inter-trial dimensional change. To examine this possibility, I introduced an analysis aimed at separating the local dimensional switch cost from the CDC. In studies using a task-switching paradigm, it is known that RTs are affected by two sources (Meiran, 2000). One source is referred to a mixing cost, in which RTs on certain trials are affected by global information of an experimental block. The other source is called switching cost; here RTs in a given trial are affected by the level of consistency between a current a task in the previous trial. A similar logic holds for this experiment. That is, the ITF is considered similar to the task-switching cost. However, the CDC in the original analysis should be influenced by these two sources. Thus, in addition to the analysis of overall RT used in previous studies (e.g., Müller et al., 1995), this study examined only the trials that followed the same target-defining feature, which allows for elimination the effect of task-switch cost caused by trial *n* − 1. If the CDC is largely affected by the task-switching cost, then the CDC effect should be eliminated by this analysis.

The present study examines the effect of dimensional knowledge on attentional focusing to a target location using response congruency effect as an index. If dimensional knowledge is effective under the search strategy manipulation, the CDC should emerge. If dimensional knowledge leads to efficient attentional guidance to the target location, then response congruency effect should be reduced when the target dimension is known (in the within-dimension condition) relative to when the target dimension is varied across trials (in the cross-dimension condition).

In addition, the present study aimed to further clarify the locus of ITF by focusing on the response congruency effect. If the ITF depends on processing in the response selection stage (e.g., Cohen and Magen, 1999; Feintuch and Cohen, 2002; Mortier et al., 2005; Theeuwes et al., 2006), where focal attention acts only to select a target, then no response congruency effect is predicted. On the other hand, recent studies using event-related potentials have shown that the latency and amplitude of a component, referred to as N2pc, is correlated with ITF in a compound search task (Töllner et al., 2010). Since N2pc is known to reflect the focal-attention selection processes, it can be anticipated that such processes will mediate the ITF in a compound search task. This finding predicts the modulation of the response congruency effect. More specifically, if repetition of a target-defining dimension facilitates selection of a target in a compound search, then the response congruency effect should decrease in feature-repetition trials, relative to feature-change trials of a target. In any case, the response congruency effect should provide information about the ITF mechanism.

## Experiment 1

Experiment 1 was designed to examine two issues. First, one aim was to replicate, using different stimuli, previous results (e.g., Kumada, 2001) that have shown the absence of both CDC and ITF in a compound search task. Second, the present experiment explored whether prior-knowledge of target-defining features affects attentional focusing to targets indexed by the response congruency effect, even in the absence of CDC and ITF.

Experiment 1 uses a compound search task that incorporates two main variables: dimension blocking conditions (within vs. crossed) and target attribute congruency (congruent, incongruent, neutral). Main effects on RT for the dimension variable permit assessment prior-knowledge, indexed by CDC, whereas an interaction of congruency with condition permits localization of any influences of prior-knowledge on focal attending. Additionally, the influence of immediately preceding information within a block, especially in cross-dimension conditions, is assessed using trial-to-trial analyses.

### Methods

#### Participants

Eighteen undergraduate students (8 males and 10 females; 19–23 years of age) participated as paid volunteers. All had normal or corrected-to-normal visual acuity and normal color vision.

#### Apparatus

Visual stimuli were generated and controlled by a computer (Apple Power Macintosh) and presented on a 17-inch color CRT display (Sony, high-resolution color display). Experimental presentations were controlled by a Matlab program using the Psychophysics Toolbox extensions (Brainard, 1997; Pelli, 1997).

#### Design

The experimental design was a 2 × 2 × 3 repeated measures factorial with two levels of dimensional blocking (within and cross), two levels of target dimension (color and shape) and three levels of congruency of target attributes (congruent, incongruent, and neutral).

#### Stimuli

Figure 1 shows sample displays. Stimulus elements were square (18 mm on a side), circle (20 mm in diameter), or pentagon (12 mm on a side). Circles were either yellow, yellowish-red, or yellowish-green and the squares and pentagons were always yellow. Eight elements were placed on an imaginary circle (the radius of 41 mm, 8.2°) on black background, with equal spacing. A target display consisted of seven distractors (yellow circles) and one target. There were four types of target displays with respect to the defining-feature of a target: a yellowish-red circle, yellowish-green circle, yellow square, or yellow pentagon. The former two targets were defined by a difference in color, and the latter two targets were defined by shape differences relative to distractors. Each element contained a gray letter (E, H, or S) composed by line segments of equal lengths. Letters within distractors for given array were always identical (i.e., always E, H, or S). A letter within a target could be (equally often) an H or S. There were three conditions with respect to the congruency of letters in distractors with a letter in a target in each target display. In the congruent condition, a letter in a target was the same as letters in distractors (i.e., all Hs or all Ss; see Figures 1A,C). In the incongruent condition, a target letter was different from distractor letters, but all distractor letters could be a target letter (i.e., an H with Ss or an S with Hs, for a target with distractors; see Figures 1B,D). In the neutral condition, distractor letters were Es. Equal number of trials was presented for each congruency condition.

One experimental block contained two types of targets with equal number of trials (96 trials). In each block, two types of targets were presented in a random order. Four conditions of target definition were given by combination of targets presented in one block: within-color, within-shape, and two cross-dimensions (yellowish-red and pentagon, and yellowish-green and square). In the within-color condition, a target was either a yellowish-red circle or a yellowish-green circle. In the within-shape condition, a target was either a yellow square or a yellow pentagon. In the cross-dimension (yellowish-red and pentagon) condition, a target was either a yellowish-red circle or a yellow pentagon. In the cross-dimension (yellowish-green and square) condition, a target was either a yellowish-green circle or a yellow square.

#### Procedure

Participants were seated 57 cm from the CRT display. The sequence of each trial was as follows. First, a small white dot was presented as a fixation point in the center of the display for 1500 ms. A stimulus display was presented immediately following the offset of the fixation point; it remained exposed until the participant responded. The time from display onset to response initiation was measured as RT. Before the initiation of each block, possible targets in the block were verbally informed to participants by an experimenter, but no explicit instruction about search strategy was given. The participants' task was to search for a singleton target with respect to the color or shape of items and to respond to the letter in the target as quickly and accurately as possible by pressing one of two keys. One key was assigned to the left index finger for a letter H; another key was assigned to the right index finger for a letter S. The participants received four blocks of the within-color condition, four blocks of the within-shape condition, four blocks of the cross-dimension (yellowish red and pentagon) condition and four blocks of the cross-dimension (yellowish-green and square) condition. The order of presentation of 16 blocks was counterbalanced across participants. The first block of each condition was discarded as a practice block.

### Results

The RT outliers were discarded using a modified recursive outlier elimination procedure (Van Selst and Jolicoeur, 1994) that was separately calculated for cells of dimension blocking × target × response congruency. This resulted in the removal of 2.1% of all observations. Table 1 shows the mean correct RTs. Correct RTs were entered in to a Three-Way analysis of variance (ANOVA) with dimension blocking (within, cross), target (color, shape), and response congruency (congruent, incongruent, neutral) as main factors. Main effects of target and response congruency were significant, *F*_(1, 17)_ = 72.30, *p* < 0.0001, η^2^_p_ = 0.81; *F*_(2, 34)_ = 42.10, *p* < 0.0001, η^2^_p_ = 0.72, respectively. A target × response congruency interaction was also significant, *F*_(2, 34)_ = 18.18, *p* < 0.0001, η^2^_p_ = 0.95. The main effect of the dimension blocking and all interactions with the dimensional blocking were not significant, *p*s > 0.128. Importantly, there was no dimension blocking × response congruency interaction (see response congruency effects shown in the bottom row of Table 1). The target × response congruency interaction indicated that the response congruency effect was larger in the color target trials (43 ms) than in the shape target trials (19 ms), although the response congruency effect was significant in both trials (*p*s < 0.001).

**Table 1:** **RTs (in milliseconds) and standard errors (SEs, in parentheses) in Experiment 1**.

	**Color target**			**Shape target**		
	**Within**	**Cross**	**CDC(SE)**	**Within**	**Cross**	**CDC(SE)**
Congruent (C)	559	573	13 (7.1)	620	627	7 (5.9)
Neutral	585	597	12 (7.0)	634	636	2 (4.6)
Incongruent (IC)	602	617	15 (6.4)	643	642	–1 (4.4)
IC-C (SE)	43 (3.5)	44 (5.1)	–	23 (5.2)	15 (3.8)	–

Table 2 shows error rates. Error rates were entered into a Three-Way ANOVA with dimension blocking, target, and response congruency as main factors. The main effects of target and response congruency were significant, *F*_(1, 17)_ = 10.42, *p* = 0.0049, η^2^_p_ = 0.38; *F*_(2, 34)_ = 24.81, *p* < 0.0001, η^2^_p_ = 0.59, respectively. A target × response congruency interaction was also significant, *F*_(2, 34)_ = 8.2, *p* = 0.0013, η^2^_p_ = 0.32. Error rate was larger in the incongruent than in the congruent trials, especially for shape target trials. Neither the main effect of dimension blocking, nor other interactions were significant, *p*s > 0.196. The results are consistent with those of RTs, suggesting that there was no speed-accuracy tradeoff.

**Table 2:** **Error rates (in percentage) in Experiment 1**.

	**Color target**		**Shape target**	
	**Within**	**Cross**	**Within**	**Cross**
Congruent	2.0	2.3	3.2	4.2
Neutral	3.9	3.3	3.6	4.2
Incongruent	6.6	6.1	6.0	5.2

For an ITF analysis involving feature and response consistencies of targets in consecutive trials in the cross-dimension condition, RTs on trial *n* conditional on trial *n* − 1 condition were examined according to three categorical distinctions: first, the target dimension (color and shape) in trial *n;* second, response congruency in trial *n* (congruent and incongruent); third, target dimensional sequence on trial *n* − 1 relative to trial *n* (same and different dimension). In this analysis, trials involving a neutral condition, in which distractors contained a letter E, were excluded. In addition, only RTs of correct responses on trial *n* that followed a correct response on trial *n* − 1 were analyzed. RTs were subjected to a Three-Way ANOVA using the above three factors.

Table 3 shows the result of inter-trial analysis. Results of this analysis show that all main effects were significant, target, *F*_(1, 17)_ = 100.30, *p* < 0.0001, η^2^_p_ = 0.86; dimensional sequence, *F*_(1, 17)_ = 5.12, *p* = 0.0371, η^2^_p_ = 0.23; response congruency, *F*_(1, 17)_ = 46.45, *p* < 0.0001, η^2^_p_ = 0.74. A target × dimensional sequence interaction and a target × response congruency interaction were both significant; *F*_(1, 17)_ = 10.62, *p* = 0.0046, η^2^_p_ = 0.38; *F*_(1, 17)_ = 32.26, *p* < 0.0001, η^2^_p_ = 0.65, respectively. Other interactions, except for those mentioned above, were not significant, *p*s > 0.299. The target × response congruency interaction was evident in the RT analysis of trial *n* (shown in Table 1). The significant target × dimensional sequence interaction showed that the effect of dimensional sequence was larger in color target trials [15.4 ms of the effect, *F*_(1, 17)_ = 12.07, *p* = 0.0029, η^2^_p_ = 0.42, by a simple main effect analysis] than in shape target trials (−0.6 ms, *p* = 0.871).

**Table 3:** **Mean correct reaction times in trial *n* as a function of the dimensional sequence of target-defining feature with trial *n* − 1 and the response congruency of trial *n* in Experiment 1**.

	**Within**						**Cross**					
	**Color**			**Shape**			**Color**			**Shape**		
	**Congruency**			**Congruency**			**Congruency**			**Congruency**		
	**C**	**IC**	**IC-C**	**C**	**IC**	**IC-C**	**C**	**IC**	**IC-C**	**C**	**IC**	**IC-C**
**DIMENSIONAL SEQUENCE**												
Same	558	601	43	620	641	21	566	605	39	626	639	13
Different	559	602	43	617	639	22	577	625	48	624	640	16
ITF (SE)	1 (3.7)	1 (4.1)		−3 (4.7)	−2 (3.8)		11 (4.2)	20 (5.2)		−2 (5.2)	1 (5.8)	

*Standard errors (SEs) are shown in parentheses*.

RTs in the within-dimension condition were subjected to the same inter-trial analysis as RTs in the cross-dimension condition that took the feature sequence of consecutive trials, instead of the dimensional sequence into account. A Three-Way ANOVA was performed with target, feature sequence, and response congruency of trial *n* as main factors. The main effects of target and response congruency were significant, *F*_(1, 17)_ = 32.45, *p* < 0.0001, and η^2^_p_ = 0.66; *F*_(1, 17)_ = 58.22, *p* < 0.0001, η^2^_p_ = 0.77, respectively. The target × response congruency interaction was also significant, *F*_(1, 17)_ = 9.74, *p* = 0.0062, η^2^_p_ = 0.36. Neither the main effect of feature sequence nor any of the interactions involving the factor were significant (*p*s > 0.369).

In order to eliminate the effect of the inter-trial dimensional switch in CDC, the correct RT in only the trials preceded by the same target feature was extracted and averaged for the within-dimension and cross-dimension conditions. Mean RTs in only the feature-repetition trials were subjected to a Three-Way ANOVA with dimension blocking and target and response congruency (of trial *n*) as factors. In this analysis, the main effect of dimension blocking was not significant (*p* = 0.696), whereas the other two main effects were significant; target, *F*_(1, 17)_ = 56.72, *p* < 0.0001, η^2^_p_ = 0.77, and response congruency, *F*_(1, 17)_ = 46.96, *p* < 0.0001, η^2^_p_ = 0.73. The target × response congruency interaction was also significant, *F*_(1, 17)_ = 13.76, *p* = 0.0017, η^2^_p_ = 0.45, however, none of the other interactions involving dimensional blocking were significant (*p*s > 0.263). The result of this analysis was consistent with that of the analysis shown above that used all the RT data.

### Discussion

A lack of CDC due to dimension blocking reveals that responses to targets in the within-dimension block were not faster than response to targets in the cross-dimension block. This result replicates results of a previous study (Kumada, 2001). That is, in the compound search tasks, a CDC does not occur.

In the CDC analysis, when the inter-trial switch cost caused by dimensional switching in consecutive trials was eliminated using data of only the feature-repeat trials, the result was consistent with the analysis using all the data. There was no CDC in the additional analysis, indicating that the lack of a CDC effect was not due to inflation caused by dimensional switching.

In this study, a response congruency effect between targets and distractors was used as an index of the efficiency of spatial selection of a target. As was expected, no interaction of the response congruency effect with dimension blocking condition emerged. Even when the defining-feature dimension of targets was known prior to target presentation (in the within-dimension condition), the spatial selection of targets was as inefficient as in conditions when a target dimension was unknown in advance (in the cross-dimension condition). The dimensional knowledge could not be used for efficient selection of a target for preventing response interference from flanker distractors.

Analyzing the dimensional sequence of targets in consecutive trials indicated an inter-trial dimensional effect for only the color targets in trial *n*. However, the response congruency effect was not modulated by the dimensional repetition of targets. The congruency effect in the same dimensional sequence was equivalent to that in the different dimensional sequence, even when RTs to targets were faster on trials with repeated color targets in consecutive trials. Therefore, the resulting facilitation could not be attributed to efficient focusing of attention to the target. This indicates that dimensional weighting evoked by the inter-trial dimensional sequence did not facilitate focusing attention to a target location.

An additional experiment was conducted to examine whether a CDC emerges when displays used in Experiment 1 are presented in a simple target detection task. This experiment was required in order to assure that the lack of a prior-knowledge impact in Experiment 1 was not due to specific properties of displays used in Experiment 1. In addition to trials that always contained a target, as in Experiment 1, trials were presented in which the display contained no singleton target (in target-absent trial) in this experiment. Participants responded to the presence or absence of an odd-ball target with respect to the target-defining features. Results of this experiment reveal a clear CDC as well as the ITF. The CDC was found even when only the trials following the same target-defining feature were examined. However, the CDC effect was significantly reduced relative to trials that did not take the dimensional, or feature sequence (i.e., a conventional CDC analysis) into account. Although the CDC effect was 16 ms in color target trials and 31 ms in shape target trials in the conventional CDC analysis, it was 7 ms in color target trials and 11 ms in shape target trials in the analysis using only dimensional, or feature repeated trials. This additional experiment established that the lack of the CDC observed in Experiment 1 was not due to an artifact of the reported attributes of a target and it was concluded that the lack of CDC in Experiment 1 was not caused by the displays used in that experiment.

## Experiment 2

In Experiment 2, I hypothesize that CDC and ITF will emerge in a compound search task when participants adopt the singleton detection mode. To test this hypothesis, participants' search mode was manipulated by varying the number of targets in search displays. As in Experiment 2 of Bacon and Egeth (1994), the number of targets presented in a given display varied between 1 and 3. Given this manipulation, a strategy of searching for a singleton cannot be effectively deployed because the target is not always a singleton. Accordingly, if a participant in Experiment 1 adopted the singleton detection mode in a compound search task and this adopted mode resulted in elimination of the CDC and the ITF, then the manipulation of search mode in Experiment 2 should systematically affect the CDC and the ITF. In particular, a main effect of dimension blocking, revealing a CDC, is predicted in this experiment where participants are putatively prevented from using the singleton detection mode.

### Methods

#### Participants

Sixteen undergraduate students (9 males and 7 females, 18–24 years of age) participated as paid volunteers. All had normal or corrected-to-normal visual acuity and normal color vision.

#### Apparatus and stimuli

Apparatus and stimuli were identical to those of Experiment 1 with the following two exceptions.

First, the number of targets in target displays varied from 1 to 3. Displays used in one-target trials were identical to those in Experiment 1. For two-target trials, one of the seven distractors in one-target displays was replaced by a target. For three-target trials, one of the six distractors in two-target displays was replaced by a target. All targets on trials with displays of two-target or three-targets were identical. Participants were informed that all targets, within a given display, had the same reported letter. The three levels of target number (1, 2, and 3 targets) occurred equally often within each experimental block of trials. That is, the number of targets could vary within a given block.

Second, only congruent trials and incongruent trials were presented; neutral trials used in Experiment 1 were not included.

#### Procedure

The procedure was the same as used in Experiment 1.

### Results

RT outliers were excluded from analyses using same procedure as in Experiment 1. This resulted in the removal of 2.3% of all the observations. Table 4 shows the mean correct RTs. Correct RTs were subjected to a Four-Way ANOVA with dimension blocking, target, the number of targets, and response congruency as main factors. Of particular interest is the finding that dimensional blocking exerted a significant effect on RTs with participants responding more slowly in cross-dimension conditions than in within-dimension conditions, *F*_(1, 15)_ = 6.69, *p* = 0.0206, η^2^_p_ = 0.31. In addition, main effects of target, number of targets, and response congruency were also significant with *F*_(1, 15)_ = 58.32, *p* < 0.0001, η^2^_p_ = 0.80; *F*_(2, 30)_ = 95.27, *p* < 0.0001, η^2^_p_ = 0.86; *F*_(1, 15)_ = 105.93, *p* < 0.0001, η^2^_p_ = 0.88, respectively. The dimension blocking × response congruency interaction, the target × number of target interaction, the target × response congruency interaction, and the number of target × response congruency interaction were significant with *F*_(1, 15)_ = 7.92, *p* = 0.0131, η^2^_p_ = 0.35; *F*_(2, 30)_ = 9.57, *p* = 0.0006, η^2^_p_ = 0.39; *F*_(1, 15)_ = 6.18, *p* = 0.0252, η^2^_p_ = 0.29; *F*_(2, 30)_ = 5.5, *p* = 0.0066, η^2^_p_ = 0.28, respectively. Other interactions were not significant. The dimension blocking × response congruency interaction showed that the congruency effect was larger in the within-dimension block (40 ms) than in the cross-dimension block (32 ms), both *p*s < 0.0001 by a simple main effect analysis. The target × the number of targets interaction showed that RTs increased with the number of targets, and this tendency was larger for shape targets than for color targets. In addition, a significant interaction of target × response congruency revealed that the response congruency effect was larger in the color target (42 ms) than in the shape target (30 ms). A number of target × response congruency interactions showed that the response congruency effect increased with the number of targets (31, 36, and 41 ms, respectively for one-, two- and three-target condition).

**Table 4:** **RTs (in milliseconds) and standard errors (SEs, in parentheses) in Experiment 2**.

	**Color target**			**Shape target**		
	**Within**	**Cross**	**CDC (SE)**	**Within**	**Cross**	**CDC (SE)**
**TARGET = 1**						
Congruent	556	578	22 (7.0)	626	645	16 (8.3)
Incongruent	596	613	17 (6.9)	657	667	10 (9.1)
IC-C (SE)	39 (5.0)	35 (4.6)		27 (7.2)	22 (4.0)	
**TARGET = 2**						
Congruent	557	583	26 (5.0)	640	662	22 (10.6)
Incongruent	606	625	20 (8.2)	673	681	8 (7.5)
IC-C (SE)	49 (5.7)	43 (4.6)		33 (8.1)	19 (4.9)	
**TARGET = 3**						
Congruent	573	602	29 (8.5)	669	679	10 (6.9)
Incongruent	624	641	17 (7.0)	712	712	0 (11.9)
IC-C (SE)	51 (6.0)	39 (5.7)		43 (6.8)	33 (9.0)	

Table 5 shows error rates. Error rates were subjected to a Four-Way ANOVA with dimension blocking, target, the number of targets, and response congruency as main factors. The main effects of the number of targets and response congruency were significant, *F*_(2, 30)_ = 50.53, *p* < 0.0001, η^2^_p_ = 0.77; *F*_(1, 15)_ = 38.52, *p* < 0.0001, η^2^_p_ = 0.72, respectively. The dimension blocking × response congruency interaction and the target × the number of targets × response congruency interaction were both significant with *F*_(1, 15)_ = 6.08, *p* = 0.0262, η^2^_p_ = 0.29; *F*_(2, 30)_ = 3.86, *p* = 0.0323, η^2^_p_ = 0.20, respectively. Other interactions were not significant. These trends were not compatible with those of RTs and this suggests the absence of speed-accuracy trade-offs.

**Table 5:** **Error rate (in percentage) in Experiment 2**.

	**Color target**		**Shape target**	
	**Within**	**Cross**	**Within**	**Cross**
**TARGET = 1**				
Congruent	1.6	2.6	1.6	1.9
Incongruent	3.4	2.9	3.1	2.7
**TARGET = 2**				
Congruent	1.8	2.2	2.5	3.8
Incongruent	4.5	3.3	4.2	3.2
**TARGET = 3**				
Congruent	3.2	3.0	3.5	3.9
Incongruent	5.1	5.5	7.2	7.0

In the above-mentioned Four-Way ANOVA of RTs, the number of targets factor interacted with other factors in several ways. However, the result was difficult to interpret compared to that of Experiment 1, in which the number of targets were not manipulated. In subsequent analyses of Experiment 2, RTs in trials with only one target were analyzed. First, the CDC was examined using a Three-Way ANOVA with dimension blocking, target, and response congruency as main factors. All three main effects were significant; dimension blocking, *F*_(1, 15)_ = 6.29; *p* = 0.0241, η^2^_p_ = 0.30; target, *F*_(1, 15)_ = 41.27; *p* < 0.0001, η^2^_p_ = 0.73; and response congruency, *F*_(1, 15)_ = 70.07; *p* < 0.0001, η^2^_p_ = 0.84, whereas none of the interactions were significant (*p*s > 0.055).

Inter-trial effects of targets were analyzed both for within-dimension and cross-dimension blocks. As shown in Table 6, in cross-dimension blocks, RTs in trial *n* with only one target were categorized by the target feature in trial *n*, the congruency of trial *n* and the dimensional (or feature) sequence of a target feature in trial *n* with that of trial *n* − 1. The number of targets displayed on trial *n* − 1 and the response sequence in consecutive trials were not taken into account. A Three-Way ANOVA was performed with target, dimensional sequence, and response congruency (of trial *n*) as main factors. All main effects were significant; target, *F*_(1, 15)_ = 37.07, *p* < 0.0001, η^2^_p_ = 0.71; dimensional sequence, *F*_(1, 15)_ = 9.46, *p* = 0.0077, η^2^_p_ = 0.39; and response congruency, *F*_(1, 15)_ = 70.79, *p* < 0.0001, η^2^_p_ = 0.82. Only the target × response congruency interaction was significant, *F*_(1, 15)_ = 5.39, *p* = 0.0347, η^2^_p_ = 0.26, whereas none of the other interactions were significant (*p*s > 0.243). The main effect of the dimensional sequence revealed that there was a significant ITF effect for both color target trials (14 ms of the effect) and for shape target trials (14 ms of the effect).

**Table 6:** **Mean correct reaction times in trial *n* as a function of the dimensional sequence of target-defining feature with trial *n* − 1 and the response congruency of trial *n* in Experiment 2**.

	**Within**						**Cross**					
	**Color**			**Shape**			**Color**			**Shape**		
	**Congruency**			**Congruency**			**Congruency**			**Congruency**		
	**C**	**IC**	**IC-C**	**C**	**IC**	**IC-C**	**C**	**IC**	**IC-C**	**C**	**IC**	**IC-C**
**DIMENSIONAL SEQUENCE**												
Same	551	597	46	623	654	31	576	603	27	638	661	23
Different	563	595	32	637	660	23	582	624	42	653	674	21
ITF (SE)	12 (7.8)	–2 (6.9)		14 (10.8)	6 (8.9)		6 (7.6)	21 (5.4)		15 (6.9)	13 (6.9)	

*Standard errors (SEs) are shown in parentheses*.

The same analysis as in the cross-dimension condition was conducted for the within-dimension condition. In this analysis, the feature sequence of targets in consecutive trials was taken into account, instead of the dimensional sequence. A Three-Way ANOVA with target, feature sequence and response congruency as main factors indicated that the main effects of target and response congruency were significant, *F*_(1, 15)_ = 36.42, *p* < 0.0001, η^2^_p_ = 0.71; and *F*_(1, 15)_ = 54.83, *p* < 0.0001, η^2^_p_ = 0.79, respectively. The main effect of feature sequence was marginally significant; *F*_(1, 15)_ = 3.21, *p* = 0.0931, η^2^_p_ = 0.18, whereas none of the interactions were significant (*p*s > 0.183).

In order to eliminate the effect of ITF in CDC, a correct RT only in a trial with one-target preceded by the same target feature or dimension was extracted, and then averaged both for the within-dimension condition as well as the cross-dimension condition. Mean RTs only for feature-repetition trials were subjected to a Three-Way ANOVA with dimension blocking, target, and response congruency as factors. In this analysis, although main effects of target and response congruency were significant; *F*_(1, 15)_ = 44.21, *p* < 0.0001, η^2^_p_ = 0.75; and *F*_(1, 15)_ = 41.26, *p* < 0.0001, η^2^_p_ = 0.73, respectively, the main effect of dimension blocking was not significant (*p* = 0.141). Moreover, the dimension blocking × response congruency interaction was significant, *F*_(1, 15)_ = 6.76, *p* = 0.0201, η^2^_p_ = 0.31. A simple main effect analysis revealed that the effect of dimension blocking was significant only in the response congruent trials [20 ms of CDC, *F*_(1, 15)_ = 6.18, *p* = 0.0252, η^2^_p_ = 0.29], but not in the response incongruent trials (6 ms of CDC, *p* = 0.537). The response congruency was significant both for within-dimension [39 ms of response congruency, *F*_(1, 15)_ = 36.49, *p* < 0.0001, η^2^_p_ = 0.71] and cross-dimension conditions [25 ms, *F*_(1, 15)_ = 27.67, *p* = 0.0001, η^2^_p_ = 0.64].

### Discussion

The significant impact of dimension blocking in this experiment, which showed that RTs to targets in the within-dimension block were faster than those in the cross-dimension block for both color and shape targets, is consistent with the prediction that an CDC can occur even in a compound search task. In particular, this effect can be attributed to the fact that participants did not adopt the singleton detection mode.

This interpretation is supported the fact that these findings are not inconsistent with those of Experiment 1 where participants also performed a compound search task. The critical difference between this experiment and previous ones (including Experiment 1) entailed manipulation of participants' mode of target search. In previous studies, targets in search displays have been typically assumed a singleton status; therefore it is likely that participants adopted a singleton search mode in the cross-dimension condition, and in the within-dimension condition as a default even though feature knowledge is available. On the other hand, in this experiment, targets were not always singletons, thus precluding a singleton search mode. The present result supported the hypothesis that adopting the singleton detection mode eliminated top-down control for visual search based on prior knowledge of target-defining feature dimensions.

A related issue raised by findings of Experiment 2 concerns response congruency. In this experiment the analysis of overall RTs and dimensional, or feature repeated trials indicated that the response congruency effect was larger in within-dimension block conditions than in cross-dimension block conditions. This is inconsistent with the prediction that dimensional knowledge is primarily effective for attentional guidance to the target location; it is also inconsistent with previous studies showing that the response congruency effect is reduced when a target is dissimilar to distractors (e.g., Baylis and Driver, 1992). To explain this unexpected outcome, I consider another factor that may modulate the response congruency effect. The response congruency effect is larger in the color target trials than in the shape target trials in spite of the fact that that overall RTs were faster in the color target trials than in shape target trials. Since the distractors were always constant over trials and also the reported attributes were the same in both target conditions, the difference of baseline RTs can be due to the saliency of target features among distractors. Therefore, this suggests that there is a large response congruency effect when a target is salient among distractors. Previous models of dimension weighting, such as Found and Müller (1996), propose that perceptual signals in the feature processing stage are enhanced via a dimension weighting mechanism. In this experiment, the CDC was reliable, implying that the feature-based dimension weighting mechanism did function properly. Consequently, the perceptual signal of targets was enhanced via this mechanism, explaining the presence of a larger response congruency effect in the within-dimension trials. Although the functional similarity between the physical saliency of targets and their perceptual saliency was modulated by the dimensional weighting mechanism would seem to explain the apparent similarity of the patterns of interaction between target × response congruency and dimension blocking × response congruency, the reason for the larger response congruency effect when the target was salient is not fully explained. This topic is pursued further in the General Discussion.

There were significant ITFs in both color and shape targets. This should be contrasted with the result of Experiment 1, in which ITF was significant only for the color target. Adopting other strategies (e.g., a feature search mode) than the singleton detection mode affected implicit memory for target dimensions stored during consecutive trials. In this experiment, however, given sufficient ITFs in both color and shape targets, a response congruency effect was independent of inter-trial dimensional sequence. This shows that implicit dimensional information carried-over from previous trials is not useful for efficient attentional focusing to a target location. Rather, this suggests that implicit inter-trial dimensional priming is independent of control of spatial attention.

The CDC analysis indicated that the CDC effect was attenuated when the local switch cost caused by dimensional switching in consecutive trials was eliminated by using only the data in feature, or dimension-repeat trials. This pattern of results was similar to the additional experiment reported in the Discussion of Experiment 1. In Experiment 2, the main effect of dimension blocking was not significant, but it interacted with the response congruency. This indicated that the CDC was observed only in response congruent trials. On the other hand, the main effect of dimension blocking was significant in the overall RT analysis, indicating that the CDC was observed for both congruent and incongruent trials. Eliminating the dimensionally changed trials made RTs generally faster compared to the overall data for both congruent (5.1 ms of reduction) and incongruent trials (8.6 ms of reduction) in the cross-dimension block, and for congruent trials in the within-dimension block (6.5 ms of reduction). However, in incongruent trials of the within-dimension block, results of RT analysis using all the data was nearly equivalent to results of the analysis with dimensionally repeated-trials (0.9 ms of difference). This suggests that RTs in the current trial was affected by the feature sequence in consecutive trials for all trials, with the exception of incongruent trials in the within-dimension block. In fact, for congruent trials, the size of CDC in the overall analysis was equivalent to that in the analysis with dimensionally repeated-trials (19 vs. 20 ms). The reason why the feature sequence was only insensitive for incongruent trials in within-dimension blocks is unclear. It is worthwhile noting however, that the CDC effect was found when the local dimensional switch effect was discounted, at least in response congruent trials.

## Experiment 3

Experiment 2 showed that the CDC emerged when participants were forced *not* to adopt the singleton detection mode by increasing the number of targets. In Experiment 3, I examined whether the CDC can again be eliminated if participants are forced to adopt the singleton detection mode even though multiple targets are presented. In order to shift participants' search mode to singleton detection, targets were always presented in adjacent spatial positions within a circular array as “a singleton region.” Participants were informed that targets were always presented as a “clump” and were asked to search for a “singleton” region in target displays. If, in this context, participants follow instructions and adopt the singleton detection mode, then no CDC and ITF should emerge even with multiple targets.

### Methods

#### Participants

Eighteen undergraduate students (12 males and 6 females; 19–23 years of age) participated as paid volunteers. All had normal or corrected-to-normal visual acuity and normal color vision.

#### Apparatus and stimuli

Apparatus and stimuli were identical to those of Experiment 2, except that targets were always presented in adjacent locations in two-target and three-target trials. Participants were informed that targets always appeared in a clump and instructed to search for a clump of targets.

#### Procedure

The procedure was the same as used in Experiment 2.

### Results

RT outliers were excluded from the analysis by the same procedure as in Experiment 1. This resulted in the removal of 2.2% of all observations. Table 7 shows the mean correct RTs. Correct RTs were entered into a Four-Way ANOVA with dimension blocking, target, number of target and response congruency as main factors. The main effects of target, the number of targets, and response congruency were significant, *F*_(1, 17)_ = 56.09, *p* < 0.0001, η^2^_p_ = 0.73; *F*_(2, 34)_ = 55.84, *p* < 0.0001, η^2^_p_ = 0.77; *F*_(1, 17)_ = 29.38. *p* < 0.0001, η^2^_p_ = 0.63, respectively. The dimension blocking × number of target interaction, target × response congruency interaction and number of targets × response congruency interaction were significant, *F*_(2, 34)_ = 3.35, *p* = 0.0471, η^2^_p_ = 0.16, *F*_(1, 17)_ = 27.44, *p* < 0.0001, η^2^_p_ = 0.62; *F*_(2, 34)_ = 07.15, *p* = 0.0026, η^2^_p_ = 0.30, respectively. The main effect of dimension blocking and other interactions were not significant, *p*s > 0.274. The significant interaction of dimension blocking × number of target showed that RTs slowed as the number of targets increased and this trend differed as a function of the dimension blocking conditions. There was no significant effect of dimension blocking in all levels of the number of targets (*p*s > 0.275; −0.1, 5.7, and −2.6 ms, in the size of CDC, respectively for one-, two-, and three-target trials by a simple main effect analysis).

**Table 7:** **RTs (in milliseconds) and standard errors (SEs, in parentheses) in Experiment 3**.

	**Color target**			**Shape target**		
	**Within**	**Cross**	**CDC (SE)**	**Within**	**Cross**	**CDC (SE)**
**TARGET = 1**						
Congruent	581	580	−1 (5.6)	651	648	−3 (9.6)
Incongruent	605	612	7 (6.5)	664	660	−4 (7.4)
IC-C (SE)	24 (4.2)	32 (6.9)		13 (5.7)	12 (6.7)	
**TARGET = 2**						
Congruent	576	589	13 (6.5)	646	651	5 (10.2)
Incongruent	611	617	6 (5.4)	663	662	−1 (7.1)
IC-C (SE)	35 (6.5)	28 (6.2)		17 (9.2)	11 (4.6)	
**TARGET = 3**						
Congruent	593	597	4 (7.5)	667	663	−4 (9.7)
Incongruent	635	637	2 (5.2)	693	681	−12 (7.0)
IC-C (SE)	42 (6.8)	40 (8.2)		26 (7.8)	18 (6.7)	

A similar CDC analysis was performed using only the RTs in trial *n* with one-target, with target, dimensional sequence, and response congruency as main factors. Main effects of target and response congruency were significant; *F*_(1, 17)_ = 53.59, *p* < 0.0001, η^2^_p_ = 0.76; and *F*_(1, 17)_ = 30.21, *p* < 0.0001, η^2^_p_ = 0.64, respectively. In addition, the target × response congruency interaction was significant, *F*_(1, 17)_ = 13.60, *p* = 0.0018, η^2^_p_ = 0.44. However, neither the main effect of dimension blocking, nor any interactions involving the effect was significant (*p*s > 0.392).

Table 8 shows error rates. Error rates were subjected to a Four-Way ANOVA with dimension blocking, target, number of targets and response congruency as main factors. The main effects of number of targets and response congruency were both significant, *F*_(2, 34)_ = 20.35, *p* < 0.0001, η^2^_p_ = 0.54; *F*_(1, 17)_ = 19.17, *p* = 0.0004, η^2^_p_ = 0.53, respectively. The target × number of targets interaction and the target × response congruency interaction were significant, *F*_(2, 34)_ = 5.10, *p* = 0.0116, η^2^_p_ = 0.23; *F*_(1, 17)_ = 12.55, *p* = 0.0025, η^2^_p_ = 0.43, respectively. The main effects of dimension blocking and target, and other interactions except above were not significant, *p*s > 0.198. First, the error rate tended to increase with the number of targets, but the tendency was more prominent in the color target trials than in the shape target trials. Second, the error rate was larger in the congruent trials than in the incongruent trials, especially for the color target trials relative to the shape target trials. These results were consistent with those in RTs. More importantly, neither a main effect of the dimension blocking nor interactions of dimension blocking with other factors were significant, suggesting that there was no speed-accuracy tradeoff.

**Table 8:** **Error rates (in percentage) in Experiment 3**.

	**Color target**		**Shape target**	
	**Within**	**Cross**	**Within**	**Cross**
**TARGET = 1**				
Congruent	1.7	2.2	2.1	2.7
Incongruent	4.1	4.5	3.7	3.5
**TARGET = 2**				
Congruent	2.0	1.8	2.7	3.4
Incongruent	3.7	4.4	3.6	4.7
**TARGET = 3**				
Congruent	3.0	3.2	3.8	4.3
Incongruent	5.1	5.0	5.0	4.9

Table 9 shows the result of inter-trial analysis. Inter-trial effects of targets in the cross-dimension condition were analyzed as in Experiment 2, using only trials with one-target. A Three-Way ANOVA was performed with target, dimensional sequence and response congruency as main factors. All main effects were significant; target, *F*_(1, 17)_ = 72.60, *p* < 0.0001, η^2^_p_ = 0.81; dimensional sequence, *F*_(1, 17)_ = 14.52, *p* = 0.0014, η^2^_p_ = 0.46; and response congruency, *F*_(1, 17)_ = 23.26, *p* = 0.0002, η^2^_p_ = 0.58. In addition, only the target × response congruency interaction was significant, *F*_(1, 17)_ = 6.34, *p* = 0.0221, η^2^_p_ = 0.27, whereas none of the other interactions were significant (*p*s > 0.309). In particular, there was no dimensional sequence × response congruency interaction (*p* = 0.391), indicating that the response congruency effect was similar in the dimension-repeat trials (19 ms) to that found in the dimension-change trials (24 ms). Although the main effect of dimensional sequence was significant, it did not interact with other factors.

**Table 9:** **Mean correct reaction times in trial *n* as a function of the dimensional sequence of target-defining feature with trial *n* − 1 and the response congruency of trial *n* in Experiment 3**.

	**Within**						**Cross**						
	**Color**			**Shape**			**Color**			**Shape**			
	**Congruency**			**Congruency**			**Congruency**			**Congruency**			
	**C**	**IC**	**IC-C**	**C**	**IC**	**IC-C**	**C**	**IC**	**IC-C**	**C**	**IC**		**IC-C**
**DIMENSIONAL SEQUENCE**													
Same	572	598	26	640	664	24	575	600	25	641		655	14
Different	589	612	23	662	664	2	585	624	39	655		666	11
ITF (SE)	17 (4.4)	14 (7.2)		22 (8.4)	0 (7.9)		10 (6.0)	24 (7.1)		14 (8.7)		11 (4.5)	

*Standard errors (SEs) are shown in parentheses*.

A similar ITF analysis as the cross-dimension condition was performed for within-dimension conditions. All main effects were significant; target, *F*_(1, 17)_ = 34.46, *p* < 0.0001, η^2^_p_ = 0.67; feature sequence, *F*_(1, 17)_ = 15.70, *p* = 0.0010, η^2^_p_ = 0.48; and response congruency, *F*_(1, 17)_ = 22.77, *p* = 0.0002, η^2^_p_ = 0.57, whereas none of the interactions were significant (*p*s > 0.069).

Next, correct RTs only for feature repetition trials were subjected to a Three-Way ANOVA with dimension blocking, target and response congruency as main factors. Main effects of target and response congruency were significant; *F*_(1, 17)_ = 54.74, *p <* 0.0001, η^2^_p_ = 0.76; and *F*_(1, 17)_ = 33.41, *p* < 0.0001, η^2^_p_ = 0.66, respectively, whereas nether the main effect of dimensional blocking nor any of the interactions were significant (*p*s > 0.307).

### Discussion

The CDC was eliminated in Experiment 3 in any ways of analyses. This was also true when local dimensional switch cost was discounted in the analysis with feature-repeated trials or when RTs only in one-target trials were analyzed. A critical difference between this experiment and Experiment 2 involved the spatial arrangement of targets together with instructions to participants. These manipulations appeared to encourage participants to adopt the singleton detection mode in spite of the fact that multiple targets could appear in some search displays. In this respect then, these results resemble those of Experiment 1 where participants appeared to adopt the singleton search mode.

The ITF was found when participants were encouraged to adopt the singleton search mode. Furthermore, this was the case even when the CDC was eliminated by this mode manipulation. In fact, the results of Experiment 3, when compared to those of Experiment 2, indicate that the ITF shows the same range in both experiments (14 ms in Experiment 2 and 15 ms in Experiment 3). However, the CDC was largely reduced in this experiment (0 ms) relative to its value in Experiment 2 (16 ms). This experiment also yielded findings inconsistent with those of Experiment 1, in which no CDC with weak ITF were found. This suggested that mode manipulation only affects the dimension-weighting mechanism relevant for CDC, but not on the mechanism for ITF. This topic is pursued further in the General Discussion.

Consistent with Experiments 1 and 2, the response congruency did not interact with inter-trial dimensional sequence of targets, suggesting that the mechanism for attentional focusing to a target is independent of inter-trial dimensional priming. Dimension weighting based on inter-trial dimensional sequence did not affect attentional guidance to target locations.

## General discussion

In this study, the CDC was found in a compound search task when participants were forced not to adopt the singleton detection mode, by changing the context of target displays. When singleton target displays were presented in the same experimental blocks as displays containing two or three targets, the CDC was observed (in Experiment 2). However, when only singleton target displays were presented in one experimental block (in Experiment 1) or when singleton target displays were presented with multiple targets that always appeared as a clump (in Experiment 3), the CDC was eliminated. This showed that the prior-knowledge of target-defining features facilitated visual search only when participants could not adopt the singleton detection mode, and were forced to enlist a feature search mode, even though a target feature was not consistent within a block in the within-dimension condition.

The main result in this set of experiments is consistent with the hypothesis that an apparent inability of feature-based top-down control in compound search is not due to a structural limitation of visual processing. So far, elimination of dimension-weight effects in compound search tasks is typically regarded favoring impenetrability of top-down control to the first parallel stage of visual processing (e.g., Theeuwes et al., 2006). This interpretation predicts no effects of mode manipulation on the CDC, however, the present results do not support this prediction. There is a consensus that feature-based modulation occurs in an early stage of visual processing, at least when a simple search task is used (Found and Müller, 1996; Müller and Krummenacher, 2006). The present study considered whether such a modulation extends to the compound search task, given that a participant adopts a specific search strategy. For instance, a recent study, in which a participant's search strategy was manipulated by a prior cue in a trial-by-trial manner in an oculomotor capture task, showed that top-down control affects the processing of distractors at an early stage (Moher et al., 2011). Therefore, it is possible that by adopting a feature search mode, top-down control can be effective in the early processing stages in a wide range of attentional tasks.

The response congruency effect was modulated by dimension blocking of targets when the CDC was found in the overall RT analysis of Experiment 2. Also, the response congruency effect was larger in the within-dimension condition than in the cross-dimension condition. This is inconsistent with the primary prediction offered in Introduction which is based on the assumption that dimensional knowledge leads to efficient attentional guidance to the target location. Instead, it favors an alternate proposal that prior-knowledge of a target-defining feature dimension affects target detection through a reduction of spatial resolution of focal attention to a target location. So far, to the best of my knowledge, no studies have reported this form of trade-off between the feature-based control of attention and space-based control of attention in CDC. However, a related idea has been proposed by Krummenacher et al. (2009), who showed that spatial attention (referred to as space-based attention) is employed in a cross-dimension condition of a compound search task. They also argued that this space-based weighting conversely reduced resources for dimension-based weighting in this task due to a “competitive relationship” between these two weighting mechanisms for allocation of limited pool of attentional (weight) resources. Although Krummenacher et al. demonstrated a trade-off between space-based weighting and dimension-based weighting with ITFs, results of the present study can be interpreted to mean that these two weighting strategies are governed by a common mechanism.

Given the competitive mechanism between the space-based control and feature-based control, it remains difficult to explain results of the present study particularly those involving the interaction of dimension blocking × response congruency under appropriate search strategy. To reconcile these results with current models of dimension weighting, I propose that dimensionally weighted signals contain only coarse location information. There is ample evidence that the visual attention system uses coarse location information before attention is engaged (e.g., Cohen and Ivry, 1989, 1991; Tsal et al., 1995; Luck et al., 1997). I argue that spatially coarse information may be sufficient to target detection in a simple detection task because precise attentional focusing is not required in such a task. However, this is not true for a compound search task. In the latter, dimensionally weighted spatially coarse information should facilitate attentional guidance to a broader region containing a target and this can cause misguided attention, specifically attention that overlooks a finer-grained spatial location of a target. Such broader attentional focusing will enhance processing of flanker distractors, and consequently yields a response congruency effect.

The coarse location coding hypothesis of dimension-weighting is consistent with the discovery of an interaction between target number and response congruency in Experiment 2. In this experiment, the response congruency effect increases as number of targets increases although overall RTs increased with the number of targets. By dimension weighting, feature information is quickly transferred to the higher level of processing, but with multiple targets in a display, the specific location to be attended is inevitably coarse. In addition, there is competition among multiple targets for response selection, because one of targets must be selected to access the reported attribute. The selection of a single target among other targets requires extra time to sort through competing targets. This is compatible with the present results that show over RTs slow and response congruency increases as the number of targets increases.

With respect to ITF over consecutive trials, the results were not consistent across experiments. The ITF was found only in color target trials in Experiment 1. On the other hand, the ITF was found for both target dimensions of color and shape in Experiments 2 and 3. Although the critical condition for emerging the ITF is not fully clear in compound search tasks, one point is relevant. On average, ITF did not directly covary with the CDC, nor did it appear related to participants' search modes. The CDC was found in Experiment 2, but not in Experiment 3 whereas ITF was clearly evident in both experiments. If ITF is mainly responsible for CDC, then the CDC should appear in both experiments. This suggests that ITF was dissociable from the CDC. This is consistent with a dual mechanism account of dimension weighting (Kumada, 2001; Kumada and Hibi, 2004) in which it is assumed that CDC is mediated by an explicit weighting mechanism whereas ITF is mediated by an implicit response based on a priming mechanism (see also Rangelov et al., 2012). In addition, the present results revealed that the ITF was not affected by the participant's search mode, while the CDC was sensitive to search mode manipulations. The fact that the ITF was insensitive to the participants search mode may also be related to the implicit and automatic nature of this effect.

The present study revealed that inter-trial dimensional priming is independent of attentional focusing to targets. The response congruency effect did not interact with the ITF in the cross-dimension condition in all three experiments: the response congruency effect in the dimensional-repeat trials was equivalent to that in the dimensional-change trials even when clear ITF was found. A recent framework of MWS has argued that dimension-specific ITF involves two mechanisms, one pre-attentive and the other post-attentive (Rangelov et al., 2011a,b, 2012). However, when the flanker congruency effect is considered as a measure of attentional focusing precision, the duel ITF mechanism proposed by the MWS framework clearly does not contribute to explaining precise attentional focusing at target locations. Further investigation is needed to obtain convincing data with respect to the locus of processing on ITF.

In summary, the present study showed that dimension weighting is not always applied when dimensional knowledge is available in advance. Instead, application of the knowledge depends on the search mode that participants adopt, given by a stimulus context. When participants adopted the feature search mode, dimension weighting appears to be applied in earlier than response selection stages; moreover, it is independent of inter-trial dimensional priming. On the other hand, when participants adopted the singleton detection mode, prior dimensional knowledge is no longer effective in a target search. The singleton detection mode may be useful for detecting salient events in visual field independent of prior information biasing search targets. As a whole, our attention system is very adaptive in that it can select appropriate processing modules depending on stimulus context.

### Conflict of interest statement

The author declares that the research was conducted in the absence of any commercial or financial relationships that could be construed as a potential conflict of interest.
